# Analysis and Refactoring of Software Systems Using Performance Antipattern Profiles

**DOI:** 10.1007/978-3-030-45234-6_18

**Published:** 2020-03-13

**Authors:** Radu Calinescu, Vittorio Cortellessa, Ioannis Stefanakos, Catia Trubiani

**Affiliations:** 8 University of Paderborn, Paderborn, Germany; 9 ICREA, Open University of Catalonia, Barcelona, Spain; 10 University of York, York, United Kingdom; 11 University of L’Aquila, L’Aquila, Italy; 12 Gran Sasso Science Institute, L’Aquila, Italy

## Abstract

Refactoring is often needed to ensure that software systems meet their performance requirements in deployments with different operational profiles, or when these operational profiles are not fully known or change over time. This is a complex activity in which software engineers have to choose from numerous combinations of refactoring actions. Our paper introduces a novel approach that uses performance antipatterns and stochastic modelling to support this activity. The new approach computes the performance antipatterns present across the operational profile space of a software system under development, enabling engineers to identify operational profiles likely to be problematic for the analysed design, and supporting the selection of refactoring actions when performance requirements are violated for an operational profile region of interest. We demonstrate the application of our approach for a software system comprising a combination of internal (i.e., in-house) components and external third-party services.

## 1 Introduction

Performance antipatterns [8,31] and stochastic modelling (e.g., using queueing networks, stochastic Petri nets, and Markov models [7,16,33]) have long been used in conjunction, to analyse performance of software systems and to drive system refactoring when requirements are violated. End-to-end approaches supporting this analysis and refinement processes have been developed (e.g., [4,9,20]), often using established tools for the simulation or formal verification of stochastic models of the software system under development (SUD).

While these approaches can significantly speed up the development of systems that meet their performance requirements, they are only applicable when the SUD operational profile is known and does not change over time. Both of these are strong assumptions. In practice, software systems are often used in

* This work has been partially supported by the PRIN project "SEDUCE" n. 2017TWRCNB and by Microsoft Research through its PhD Scholarship Programme.

applications affected by uncertainty, due both to incomplete knowledge of and to changes in workloads, availability of shared resources, etc.

In this paper, we introduce a novel performance analysis and refactoring approach that addresses this significant limitation of current solutions. The new approach considers the uncertainty in the SUD operational profile by identifying the performance antipatterns present in predefined *operational profile regions*. These regions capture aleatoric and epistemic operational profile uncertainties due to unavoidable changes in the environment (e.g., workload variations) and to insufficiently measured environment properties (e.g., CPU speed), respectively.

A few existing solutions [2,11,19] employ sensitivity analysis to assess the robustness of software to variations in its operational profile. However, these solutions are not interested in major operational profile changes like our approach, and therefore focus on establishing the effect of small operational profile variations on the performance of the SUD. In contrast, our new approach provides a global perspective on the performance antipatterns associated with a wide range of operational profiles. This perspective enables software engineers to identify operational profile regions in which their SUD is likely to require refactoring, and supports the selection of suitable refactoring actions for such regions. The main contributions of this paper are:

1. We introduce the concept of a *performance antipattern profile* (i.e., a "map" showing the antipatterns present in different regions from the operational profile space of a SUD), and a method for synthesising such profiles for systems comprising a mix of internal and external software components.
2. We present a tool-supported approach that uses our performance antipattern profile synthesis method, and we define best practices for refactoring the architecture of a SUD using performance antipattern profiles.
3. We demonstrate the application of our approach for a software system comprising a combination of internal (i.e., in-house) components and external (i.e., third-party) services.

The remainder of the paper is organized as follows. Section 2 introduces a software system that we use to illustrate the application of our approach throughout the paper. Section 3 presents the new approach for the performance analysis and refactoring of software systems, and Section 4 describes its application to the service-based system from our motivating example. Section 5 compares our solution with existing approaches. Finally, Section 6 summarises the benefits and limitations of our approach, and suggests directions for future work.

## 2 Running Example

To illustrate the application of our approach, we consider a heterogeneous software system comprising both internal components and external services. We assume that the internal components are deployed on the private servers of the organisation that owns the system. As such, the architecture and resources of these components can be modified if needed. In contrast, the external services are accessed remotely from third-party providers and cannot be modified. These services can only be replaced with (or can be used alongside) other services that are functionally equivalent but may induce different performance.

## 2.1 System description

The system we use as a running example is adapted from [14], and comes from the foreign currency trading domain. The workflow implemented by this "FOREX" system is shown in Figure 1, and involves handling requests sent by currency traders. Two types of requests are possible: requests that must be handled in a so-called "expert" mode, and requests handled in a "normal" mode. The request type determines whether the system starts with a "fundamental analysis" operation or a "market watch" operation. Both of these operations use external services. "Technical analysis" is an operation provided by an internal component. This operation follows the market watch, and determines whether the trader's objectives (specified in the request) are satisfied or not. If there is a conflict between these objectives and the results of the technical analysis, then the market watch is re-executed. Furthermore, the technical analysis may return an error, i.e., an internal "alarm" operation is triggered to inform the user about the erroneous result. The optimal results of either technical or fundamental analysis (satisfied objectives/trade acceptance) lead to the execution of an external "order" operation that completes the trade, and is followed by an internal "notification" operation that confirms the successful completion of the workflow.

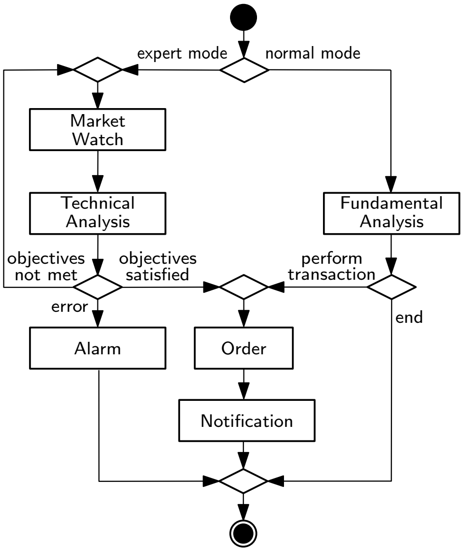

**Fig. 1.** Workflow of the foreign currency trading system (FOREX)

## 2.2 External services

For the operations executed using external services, multiple services can be used as equivalent alternatives or in some combination deemed suitable. Given $n > 1$ functionally equivalent services, three options for combining them are possible:

– *Sequential* (SEQ): first invoke service 1; if the invocation succeeds, use its response; if it fails, then invoke service 2, etc., until service $n$ is invoked, if needed.

- *Parallel* (PAR): invoke all $n$ services at once, and use the first result that comes back.
- *Probabilistic* (PROB): invoke one of the $n$ available services, selected based on a discrete probability distribution.

Therefore, we need to choose a "good" option (i.e., one that enables the system to satisfy its performance requirements) starting from information about the performance characteristics shown by each of these services, which we assume known from either the service-level agreement (SLA) published by the providers of these services, from our observations, or from both. Additionally, we assume that all these services already satisfy the functional requirements.

### 2.3 Internal components

The internal operations are executed by software components belonging to the organisation that "owns" the system, and running on their private hardware nodes/servers. We assume that technical analysis (TA) has a much more significant impact on the performance of the system compared to the other two inhouse components (alarm and notification), which require only modest resources. Consequently, it is necessary to identify possible antipattern-driven refactoring actions for the TA component, to ensure that the system operates with an optimal performance. If and when needed, the refactoring actions we consider are: (i) duplicate the TA software component and load balance the incoming requests among the two TA instances; or (ii) replace the TA instance with a faster one. These actions will increase the cost, but may be needed to satisfy the performance requirements of the system.

### 2.4 Operational profile parameters

Several parameters of the system are outside the control of its developers. These parameters represent the *operational profile* of the system. For our FOREX system, they include the probability that a user request needs expert-mode handling, and the probability of a transactions being performed after the execution of the fundamental analysis operation (cf. Figure 1). The choice of these parameter ranges reflects, for instance, the engineers' expectation about a particular deployment of the system, numerical values will be provided in Section 4.

## 3 Approach

### 3.1 Overview

As shown in Figure 2, our approach to performance analysis and system refactoring comprises five steps. Starting for an initial system design proposed by a software engineer, step 1 involves modelling the performance characteristics of the system across its entire operational profile space (i.e., for all possible

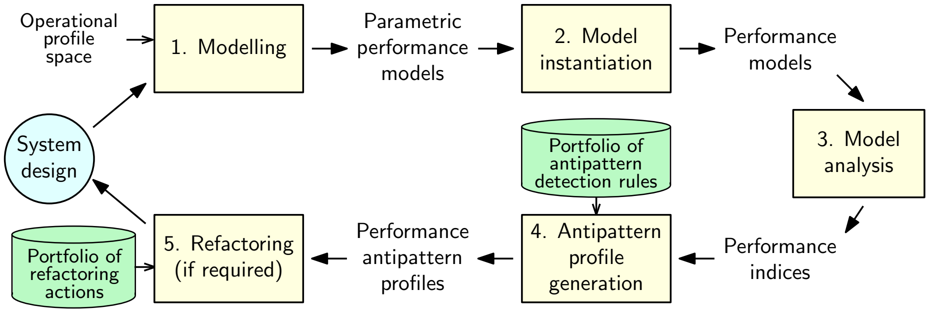

**Fig. 2.** Performance analysis and refactoring using antipattern profiles values of the operational profile parameters). As such, the performance models produced by the modelling step are *parametric models*—models containing (uninstantiated) parameters like the probabilities of receiving different types of user requests. Our approach is not prescriptive about the type of performance models that can be used in its modelling step. However, these models must be able to capture the uncertainty associated with the operational profile of the system. Therefore, in this paper we will use parametric discrete-time and continuous-time Markov chains (parametric DTMCs and CTMCs).

Step 2 of the approach instantiates the parametric performance models for combinations of parameter values covering the entire operational profile space. A suitable discretization of the continuous parameters is used for this purpose.

The performance models are then analysed in step 3 to compute the performance indices corresponding to all considered combinations of operational profile parameter values. Existing analysis tools suitable for the adopted type of performance models need to be used in this step—in the case of our DTMC and CTMC models, a probabilistic model checker such as PRISM [24] or Storm [18]([1]).

Step 4 of the approach is using the performance indices and a portfolio of antipattern detection rules to identify the performance antipatterns that occur for different combinations of parameter values. This step produces a series of maps that show the distribution of such antipatterns across the operational profile space, thus to highlight problematic (from a performance viewpoint) areas.

Finally, step 5 assesses whether refactoring actions are required, because performance antipatterns occur in regions of the operational profile space where the deployed system is expected to operate. When refactoring is required, suitable refactoring actions (selected from a repository of such actions) are used to update the system design. Updated system designs are then further evaluated through re-executing the five steps of the approach, until a design with suitable performance antipattern profiles is obtained.

[1] An estimation of the effort required to create and solve performance models is out of this paper scope, as it may depend on the application domain complexity and the analysts' expertise.

**Table 1.** Detection rule parameters.

| Variable | Scope | Description |
|---|---|---|
| InvReq | EXT/INT | Number of invocations per request |
| AvgInvReq | EXT/INT | Average number of invocations per request |
| InvTime | EXT/INT | Number of invocations per time unit |
| AvgInvTime | EXT/INT | Average number of invocations per time unit |
| ServRate | INT | Service rate |
| Util | INT | Utilization |
| AvgUtil | INT | Average utilization |
| UtilThresh | INT | Fixed utilization threshold |
| RespTime | EXT | Response time |
| AvgRespTime | EXT | Average response time |
| PathProb | EXT/INT | Probability of path execution |
| AvgPathProb | EXT/INT | Average probability of path execution |
| PathProbThresh | EXT/INT | Fixed threshold for probability of path execution |

### 3.2 Detection rules

The concept of Performance Antipattern has been introduced several years ago [31] to define bad design practices that can induce performance problems in software systems. This concept has been later formalized in First-Order Logics [17] and then employed, in the context of Software Performance Engineering processes, for the purpose of automating the detection and solution of performance problems [29].

Inspired from the formalization provided in [17], we have here bounded the detection rules of three performance antipatterns to the modeling and analysis context of this paper. This binding is indeed required for any context, due to specificities and possible limitations of the notations adopted. In our case, Markov models of service-based software systems, on one side, offer the advantage of easy deduction of stochastic indices and, on the other side, suffer of lack of separation between software and hardware parameters. The latter are in fact implicitly taken into account in execution rates of operations.

Hereafter we report the formalization of the performance antipattern detection rules that we have used in this paper, while their parameters are defined in Table 1, where we also specify whether each parameter is available for external services ('EXT'), for internal components ('INT'), or for both ('EXT/INT').

- **BLOB**

`General description`

*It occurs when a component performs most of the work of an application, thus resulting in excessive components' interactions that can degrade performance.*

`Internal components`

$(InvReq > AvgInvReq) \wedge (Util > UtilThresh) \wedge (Util > AvgUtil)$

`External components`

$InvReq > AvgInvReq$

- **CONCURRENT PROCESSING SYSTEMS (CPS)**
  `General description`
  *It occurs either when too many resources are dedicated to a component (MAX) or when a component does not make use of available resources (MIN).*
  `Internal components`
  MAX - $(Util > UtilThresh) \wedge (Util > AvgUtil)$
  MIN - $(Util < UtilThresh) \wedge (Util < AvgUtil)$
  `External components`
  MAX - $PAR\ pattern \wedge (RespTime > AvgRespTime)$
  MIN - $PAR\ pattern \wedge (RespTime < AvgRespTime)$
- **PIPE AND FILTER (P&F)**
  `General description`
  *It occurs when the slowest filter in a "pipe and filter" architecture causes the system to have unacceptable throughput.*
  `Internal and External components`
  $(InvTime > AvgInvTime) \wedge (PathProb > PathProbThresh) \wedge$
  $\wedge\ (PathProb > AvgPathProb)$

We remark that, in our context, the rules for detecting a specific antipattern on internal components may differ from the ones defined for external services. This is because the parameters available for external services are obviously more limited than those of the internally developed components. For example, the whole response time (i.e., service plus waiting time) of an external service is usually negotiated in a service-level agreement, but it is difficult to isolate the net service time contribution to it, due to lack of control on the execution platform and the amount of resources dedicated to the service by the provider. Both indices can instead be estimated for internal components. As a consequence, wherever the service time (or any derived index like utilization) appears in a detection rule, the corresponding predicate has to be skipped/modified for external services. For this reason, in our case BLOB and CPS antipatterns present different rules when applied to internal components or external services because, as reported in Table 1, utilization cannot be estimated for the latter ones. In the BLOB case, the predicates including utilization for internal components are simply skipped in the external service formulation, because no other predicate would make sense there. Instead, in the CPS case, the predicates on utilization have been replaced with similar ones on response time for external services, because the CPS definition is compliant with this modification.

We highlight that all predicates include parameters that evidently change across different areas of the system operational profile (e.g., $InvReq$, $Util$), hence we expect that the occurrences of the corresponding antipatterns vary consequently. The only exceptions are the CPS rules for external services, because their parameters and thresholds do not depend on the operational profile. Such rules refer to the response time that, for these components, is based on service level agreement, and thus it cannot vary with the operational profile. This will evidently reflect on our experimental results, where CPS on external services will appear either everywhere or nowhere in the operational profile space.

### 3.3 Synthesis of antipattern profiles

The more software applications are being used worldwide from different types of users, the more difficult is to estimate a representative average behavior of users that induces a specific operational profile. In fact, not only users can have different operational profiles depending on their locations [15], but even in the same area the users behavior can (sometime radically) change over time [23].

Nevertheless, applications should show acceptable performance across different operational profiles. A motivation for our work is that different operational profiles can induce various performance problems, for example because a higher execution frequency of a path can overload components involved in that path. Hence, the idea is that, in order to identify the most appropriate refactoring actions to apply for overcoming performance problems, these problems must be identified across different operational profiles.

In this paper, we introduce the concept of *Performance Antipattern Profile*, which is a representation of performance antipattern occurrences while varying operational profile parameters. As discussed above, different antipattern occurrences are expected to appear in different areas of an operational profile, as shown in Figure 3, where two operational profile parameters vary (from 0 to 1) on the axes, and different coloured shapes in the graph indicate the occurrences of different antipatterns. Only with this information in hand, the performance experts can suggest appropriate refactoring actions when the system falls within a certain operational profile area, or even (in a proactive way) when the system is expected to enter a specific operational profile area.

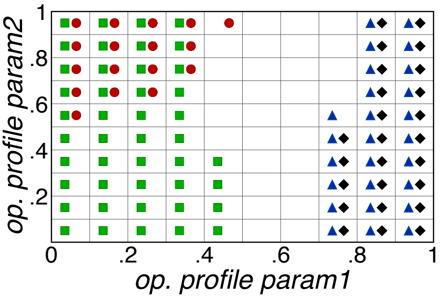

**Fig. 3.** Example of *antipattern profile*

### 3.4 Refactoring

The notational aspects outlined in the previous section for antipattern detection obviously reflect in the portfolio of refactoring actions aimed at removing performance antipatterns. In general, a refactoring action modifies some available *architectural knob* (e.g., the number of messages exchanged between two components, the list of operations provided by a component) to remove a source of the antipattern causes. The type and number of knobs depend on the adopted notation, so the portfolio of refactoring actions does the same.

Our notation distinguished between internal components and external services. The two types of system elements are characterized by a few common parameters and by parameters specific to each type (see Table 1). Therefore, our portfolio of refactoring actions is partitioned in two sets, as detailed below.

**Actions for internal components**

- `Change service rate` - The modification of a component service rate can be induced by several actions on the system, which could act on the hardware platform or on the software architecture, such as: (i) redeploy the component to a platform node with different hardware characteristics, (ii) replace some devices of the platform node where the component is currently allocated, (iii) redesign the software component so that its resource requests change, (iv) split a component into two (or more) components and re-deploy them.
- `Change number of threads` - This action is always possible where the control on the number of threads is on the designer's hands, and indeed for internal components this is guaranteed.

**Actions for external services**

- `Change pattern` - We have considered three combination patterns for external services, that are: SEQ, PAR, and PROB (see Section 2.2). They are used to combine (a subset of) the available instances of a certain external service. This action requires to modify the combination pattern, by keeping unchanged the set of combined services.
- `Change the pattern parameters` - Some patterns are regulated by parameters, in particular: PROB has a probability of each instance invocation, and SEQ has a failure probability for each instance. A change in the PROB probabilities is always feasible, because they are under full control of the designer. Instead, a change in the failure probabilities within a SEQ pattern implies that the designers are enabled for deeper modifications in the involved instances that can induce different reliability, and this is not often the case.
- `Change combination of service instances` - This action requires to replace some (or all) of service instances that are combined to provide a certain operation, by keeping unchanged the combination pattern.

Of course, the above actions can be combined together to study their joint effects on the performance improvement.

## 4 Evaluation

In this section, we first introduce the research questions that we intend to address (see Section 4.1). Thereafter, we describe the experimental scenarios (see Section 4.2) and discuss the obtained results (see Section 4.3). We finally report the threats to validity in Section 4.4. The implemented tool, the models and the experimental results are available at: https://github.com/Fase20/automated-antipattern-detection.

### 4.1 Research questions

The detection and solution of performance antipatterns largely depends on the operational profile, which is determined by the end-users behaviour, thus it can only be known after the system deployment. Naturally, some antipatterns are more affected than others by the operational profile that can have a considerable influence on the software system and, consequently, on its performance characteristics. Through our experimentation, we aim at answering the following two research questions:

- $RQ_1$: Does our approach provide insights on the performance antipattern profile of a specific design?
- $RQ_2$: Does our approach support performance-driven refactoring decisions on the basis of the performance antipattern profile?

In order to answer these questions, we apply our approach to the running example introduced in Section 2.

### 4.2 Experimental scenarios

**Table 2.** System parameters.

| Parameter | Values |
|---|---|
| ExtReqs-rate | $10s^{-1}$ |
| QueueSize | 10 |
| TA-rate | $3s^{-1}$ |
| Alarm-rate | $40s^{-1}$ |
| Notif-rate | $55s^{-1}$ |
| MW-rate | $19.92s^{-1}$ |
| FA-rate | $24.99s^{-1}$ |
| Order-rate | $19.09s^{-1}$ |
| TA-threads | 1 |

Table 2 reports the system parameters of the default configuration we have used for our experiments. It is structured in three different groups. First, system settings, i.e., *ExtReqs-rate* (rate of external requests incoming to the system), and *QueueSize* (maximum number of queueing requests). These values are both set to 10. Second, the rate of internal components and external services, e.g., $TA\text{-}rate = 3$ is the execution rate of the Technical Analysis (TA) internal component. For external services, this rate corresponds to the inverse of the response time (as explained in Section 3.2), and it was obtained through the analysis of discrete-time Markov chain (DTMC) models of the service combinations (i.e., SEQ, PAR or PROB) used for the external operations of the system. The model checker Storm was used to perform this analysis. Third, TA (as internal component) has a number of threads that is initially set to 1, but we provide a refactoring action that can change such number to modify the parallelism degree for such component.

The operational profile space of our running example (see Figure 1) is fully defined by the following branching point probabilities: (i) `pExpertMode` ($p_{EM}$), i.e., the probability of executing the workflow in expert mode; (ii) `pPerformTransaction` ($p_{PT}$), i.e., the probability of successfully performing a transaction; (iii) `pObjectivesSatisfied` ($p_{OS}$) and `pObjectivesNotMet` ($p_{ON}$), i.e., the probabilities of satisfying or not the objectives, respectively. As a consequence, $1 - (p_{OS} + p_{ON})$ is the resulting probability of an error occurring.

The experimental scenarios that we analyze in the next section include the variations of $p_{EM}$ and $p_{PT}$ within their full range $[0, 1]$ with a 0.1 step. Given the space constraints, we decided to bind $(p_{OS}, p_{ON})$ to three scenarios, namely: $\{(0.21, 0.78), (0.48, 0.01), (0.98, 0.01)\}$, which in the following we call $scenario_A$, $scenario_B$, and $scenario_C$, respectively.

We have considered the following design changes for refactoring purposes: ($R_1$) - the service rate of the TA internal component can be modified from 3 to 6 jobs per second (i.e., it becomes faster when performing computations) when TA is detected as an instance of a BLOB performance antipattern; ($R_2$) - a further thread of the TA component can be added to split the incoming load and manage users' requests, again as a solution of a BLOB performance antipattern on TA; ($R_3$) - change pattern (from SEQ to PAR) and service rate (from 50.21 to 500) of the MW external service, when MW has been detected as part of a Pipe and Filter antipattern; ($R_4$) - change service rate (from 40.02 to 400) of the FA external service while keeping the same pattern (i.e., PAR), and this is suggested as a solution of a Pipe and Filter antipattern that involves FA.

The results presented in the next section were obtained using the tool we developed to implement the analysis and refactoring process from Figure 2. This tool generates antipattern profiles using the antipattern detection rules from Section 3.2 and performance indices computed through the probabilistic model checking of a continuous-time Markov chain (CTMC) model of the entire FOREX system from Figure 1. The model checker Storm is automatically invoked by the tool for this purpose. The tool and the parametric CTMC models we used are available in our project's GitHub repository.

### 4.3 Experimental Results

In order to answer $RQ_1$, we have investigated the occurrence of performance antipatterns across different operational profiles, so to obtain performance antipattern profiles. Figures 4, 5, and 6 report the BLOB, CPS, and P&F detected antipatterns, respectively, across the operational profile space. Each figure shows the three considered scenarios for $p_{OS}$ and $p_{ON}$ and, for each scenario, $p_{EM}$ varies from 0 to 1 (with a step size of 0.1) on the x-axis , while $p_{PT}$ varies in the same range on the y-axis. Antipatterns occurring in each operational profile point are denoted by specific symbols.

We have here considered full ranges of the operational profile parameters, even though, in each instant of its runtime, the system will fall in a single point of the profile. Therefore, suitable refactoring actions depend on the area where the running system profile falls in the considered time. In particular, if it runs in an area where antipatterns do not occur, then no refactoring action is suggested.

In Figure 4(a) we can notice that in $scenario_A$ (i.e., $p_{OS} = 0.21$ and $p_{ON} = 0.78$) four different components are detected as BLOB antipatterns, specifically: (i) BLOB(FA) occurs for low values of $p_{EM}$ only (i.e., up to 0.2); as opposite, (ii) BLOB(TA) occurs for larger values of $p_{EM}$; (iii) BLOB(MW) shows a very similar behaviour with respect to BLOB(TA) except in two corner cases where it occurs alone; (iv) BLOB(Order) occurs for low values of $p_{EM}$ and high values of $p_{PT}$ only.

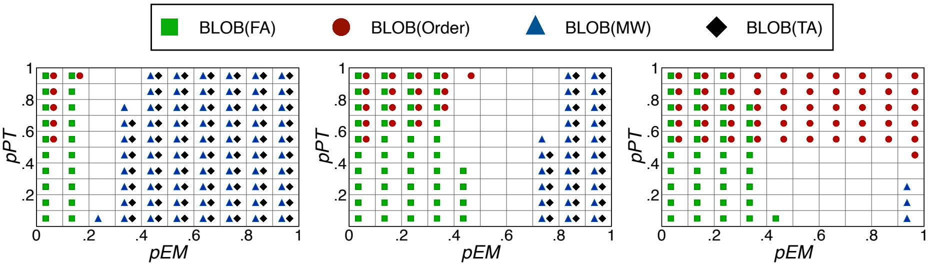

(a) $scenario_A$ (b) $scenario_B$ (c) $scenario_C$

**Fig. 4.** BLOB antipattern instances while varying operational profiles.

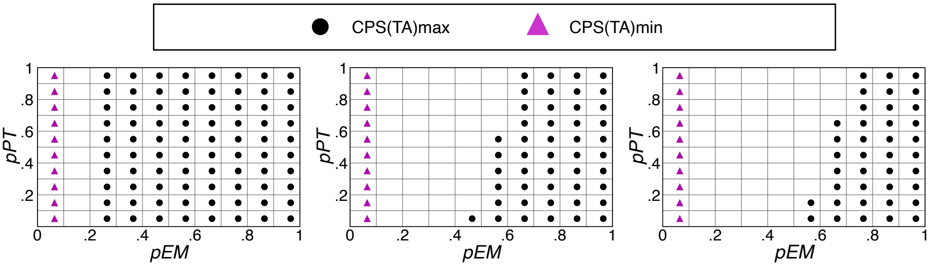

(a) $scenario_A$ (b) $scenario_B$ (c) $scenario_C$

**Fig. 5.** CPS antipattern instances while varying operational profiles.

Figure 4(b) interestingly shows that in $scenario_B$ (i.e., $p_{OS}$ = 0.48, and $p_{ON}$ = 0.01), BLOB(TA) and BLOB(MW) occur in a smaller portion of the operational profile space, i.e., the right-most side (starting when $p_{EM}$= 0.7). Also the other antipatterns are subject to the probability changes, in fact both BLOB(FA) and BLOB(Order) occur in a larger portion of the space, i.e., the left-most side (up to $p_{EM}$=0.5). This is because $scenario_B$ moves a consistent part of the workload far from the MW-TA loop, with respect to $scenario_A$.

Figure 4(c) illustrates the case of $scenario_C$ (i.e., $p_{OS}$ = 0.98, and $p_{ON}$ = 0.01), where further differences appear. In particular, BLOB(TA) antipattern does not occur anymore since the higher value of $p_{OS}$ induces less computation in TA. BLOB(MW) is confined to three cases of large $p_{EM}$ values and low $p_{PT}$ values. This is because the major load is going here to FA and Order that in fact more widely are detected as BLOB antipatterns.

Figure 5 depicts the CPS antipattern profile that, as compared to the BLOB one, does not considerably vary across different scenarios. For readability reasons, CPS(FA)min is not reported in this figure, although it occurs across the whole operational space for all the three scenarios. We recall that this is due to the CPS detection rule that takes into account the response time for external services, which does not change with users' behaviour since it is a fixed value outcoming from service-level agreements. CPS(TA)min is not affected at all by the scenario variations, as it always occurs in the same operational profile area. Instead, the CPS(TA)max instances progressively decrease when increasing $p_{OS}$. A $p_{OS}$

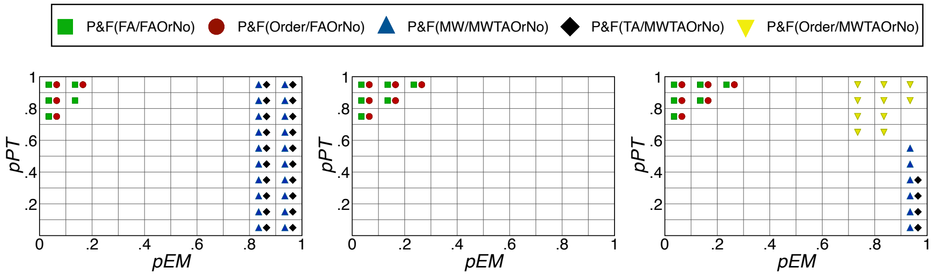

(a) $scenario_A$ (b) $scenario_B$ (c) $scenario_C$

**Fig. 6.** P&F antipattern instances while varying operational profiles.

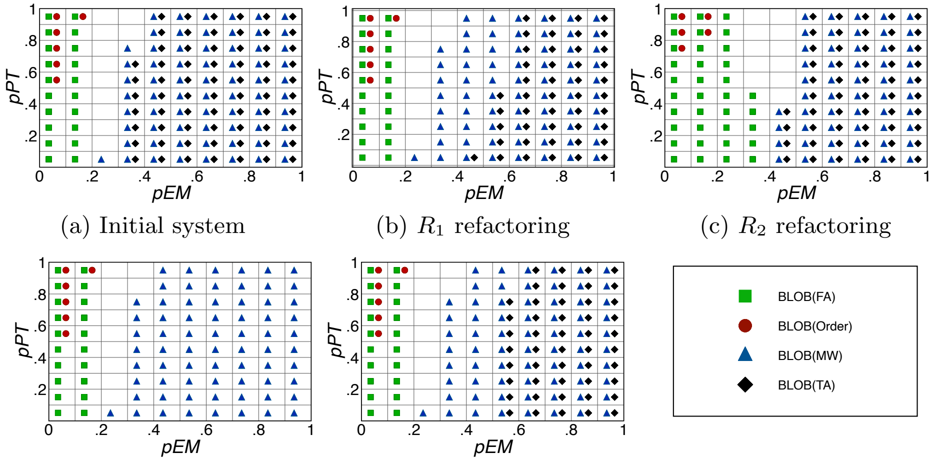

(a) Initial system (b) $R_1$ refactoring (c) $R_2$ refactoring

(d) $R_3$ refactoring (e) $R_4$ refactoring

**Fig. 7.** BLOB antipattern instances across different refactorings - $scenario_A$.

growth, in fact, relieves the MW-TA loop, thus inducing less unbalancing in its components.

Figure 6 shows the P&F antipattern profile, where the antipattern instances obviously refer to execution paths instead of single components/services. Hence, different symbols represents different paths where one of the components/services is the slowest filter. For example, MW/MWTAOrNo means that MW is the slowest filter of the MW-TA-Order-Notification path. Interesting variations of this antipattern profile appear across scenarios, again driven by variations in the operational profile parameter values.

*Summary for $RQ_1$:* Our approach provides insights on the performance antipattern profile of a specific design. In fact, we are able to identify considerable variations in the detected antipattern instances while varying the operational profile parameters.

In order to answer $RQ_2$, we have investigated the occurrence of performance antipatterns after applying refactoring actions that we have defined in Section

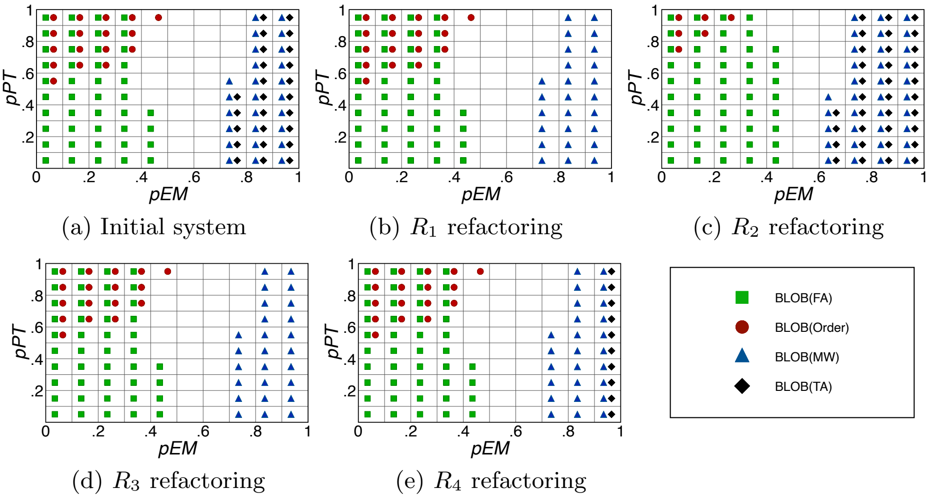

(a) Initial system (b) $R_1$ refactoring (c) $R_2$ refactoring (d) $R_3$ refactoring (e) $R_4$ refactoring

**Fig. 8.** BLOB antipattern instances across different refactorings - $scenario_B$.

4.2, across the operational profile space. The most interesting cases are discussed hereafter, and specifically: (i) Figures 7 and 8 report the BLOB refactoring effects on $scenario_A$ and $scenario_B$, respectively; (ii) Figure 9 illustrates refactorings for the CPS antipattern in $scenario_A$; (iii) Figure 10 shows the P&F refactoring effect on $scenario_C$.

In Figure 7, we can notice the following effects of refactorings actions. Upon ($R_1$) application, as expected, less BLOB(TA) instances appear because this refactoring consists of doubling the TA computation speed, while all other instances remains unvaried. ($R_2$) introduces a further TA thread and, in this case, this induces less BLOB (TA) because more quickly requests are processed by these two threads, and realistically FA becomes the overloaded one thus inducing more BLOB(FA) instances to appear. ($R_3$) modifies the rate of MW and makes it much slower, thus inducing the side effect of providing much less load to TA; in fact all the BLOB(TA) instances disappear, and all the other instances remain unvaried. ($R_4$) decreases the rate of FA and, similarly to above, it has the effect of providing less load to TA, in fact the number of BLOB(TA) instances decreases.

Figure 8 illustrates the effect of BLOB refactorings on $scenario_B$. ($R_1$) refactoring consists of making the TA component two times faster, hence the BLOB(TA) instance completely disappears from the operational space, while all the other antipatterns are not affected. ($R_2$), introduces a further TA thread, but in this case it occurs in a quite less stressed context with respect to $scenario_A$. This aspect, together with the fact that two threads allow to drop less requests, given that the queue length remains unvaried, in practice does not relieve TA itself. This is the reason for BLOB(TA) to not disappear. The decrease of BLOB(Order) instances is very likely due to the fact that, if performance indices change for some components/services, then their calculated average value change as well, hence inequalities in detection rules can change their results due

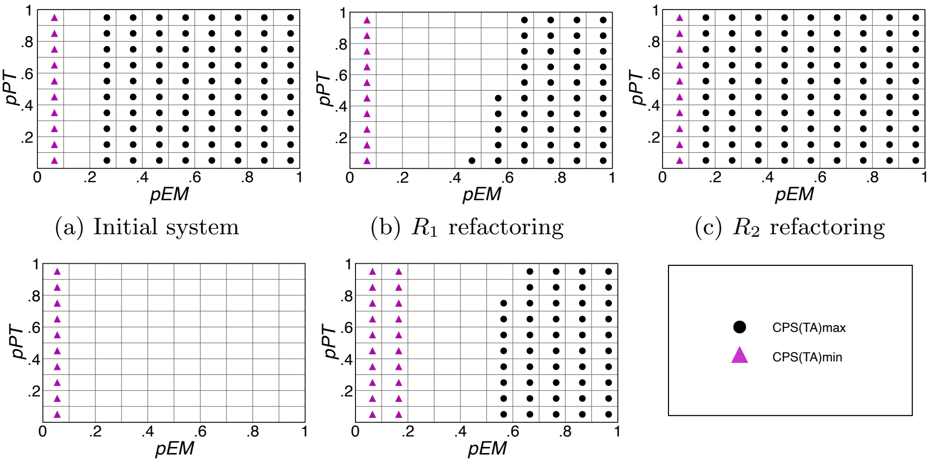

(a) Initial system (b) $R_1$ refactoring (c) $R_2$ refactoring

(d) $R_3$ refactoring (e) $R_4$ refactoring

**Fig. 9.** CPS antipattern instances across different refactorings - $scenario_A$.

to changes in the right-hand-side targets. ($R_3$), similarly to Figure 7, modifies the MW rate and makes it much slower, thus having the effect of providing much less load to TA, in fact all BLOB(TA) instances disappear. Also ($R_4$) behaves similarly to Figure 7.

Figure 9 depicts $scenario_A$ (i.e., the $p_{OS} = 0.21$ and $p_{ON} = 0.78$ case) when considering CPS antipattern instances. We recall that the detection rule for CPS on external services operates on response time values that do not change with the operational profile. This leads that CPS(FA)min occurs in the whole operational space (not only for the initial system, but also after $R_1$, $R_2$, and $R_3$ refactorings). Instead, for $R_4$ refactoring, we found CPS(FA)max always occurring, and this is due to nature of this refactoring that modifies the FA rate. For $R_3$ refactoring, besides CPS(FA)min, we also found CPS(MW)max always occurring, and this is again due to the fact that $R_3$ modifies the MW rate.

In addition to this, we can make the following specific considerations. ($R_1$), makes the TA component two times faster, hence less CPS(TA)max instances appear, as expected. ($R_2$) introduces a further TA thread but it is not beneficial for the system, in fact the number of CPS(TA)max instances increase in the operational profile space. This effect is again very likely due to the fact that, with two threads, less requests are dropped than in the one thread case. Hence the work on TA in practice increases. This apparent anomaly would be mitigated whether, in the analysis, the number of dropped requests would be considered. ($R_3$), decreases the MW rate, so it has the effect of providing less load to TA; in fact CPS(TA)max instances disappear, and (as mentioned above) a CPS(MW)max instance appears in the whole operational profile space. ($R_4$) decreases the FA rate, thus having the effect of increasing the number of CPS(TA)min instances and decreasing the CPS(TA)max ones.

Figure 10 illustrates $scenario_C$ (i.e., the $p_{OS} = 0.98$ and $p_{ON} = 0.01$ case) when considering P&F antipattern instances. Quite small variations can be ob-

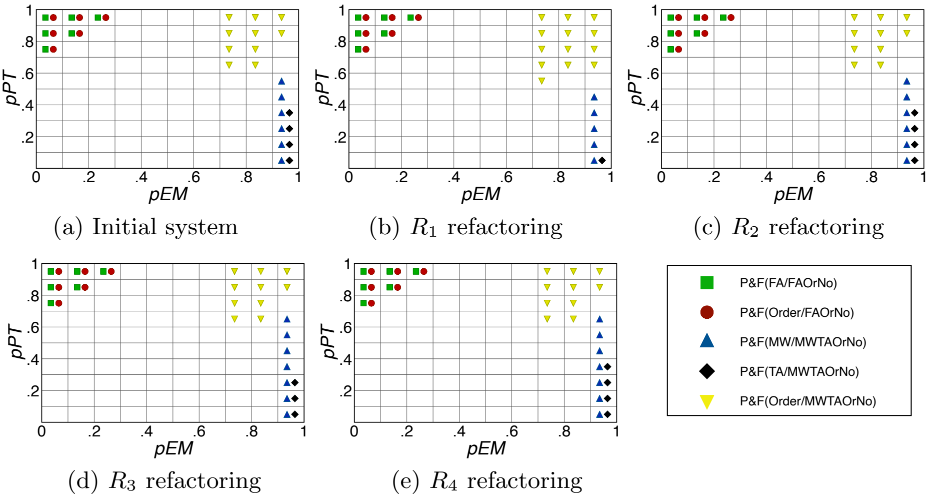

**Fig. 10.** P&F antipattern instances across different refactorings - $scenario_C$.

served here, as compared to other antipatterns and scenarios, always limitedly to single points of the operational profile space. Some specific comments follow. ($R_1$) induces less P&F instances where TA is the slowest filter and, on the same path, introduces more instances where Order is the slowest filter. This is an expected behavior due to the refactoring action that makes TA faster. ($R_2$) has no effect at all. ($R_3$) modifies the rate of MW component and makes it much slower, thus inducing less load to TA. The effect on the P&F antipattern is minimal and coherent, because one more P&F(MW) instance and one less P&F(TA) instance occur in the same path. ($R_4$) only introduces one more P&F(MW) on the same path as above, and this could be a side effect of changing the average values of performance indices.

*Summary for $RQ_2$:* The approach supports performance-driven refactoring decisions based on antipattern profiles, in that refactorings determine different effects on different regions of the operational profile space.

### 4.4 Threats to validity

*Internal validity.* In order to spot internal errors in our implementation for automatically detecting multiple performance antipatterns, we have thoroughly tested it. We verified that the detected performance antipatterns follow the given rules defined in their specification, along with the expected performance indicators. Note that the detection and solution of performance antipatterns relies on our previous experience in this domain [17], but in the future we are interested to involve external users that will be enabled to add their own rules for detection and refactoring.

*External validity.* We are aware that one case study is not enough to thoroughly validate the effectiveness of our approach. Nevertheless, several experiments have been performed beside the proposed experimental scenarios, in order to inspect the large number of variabilities in the operational profile space that may affect performance characteristics in unexpected ways. As future work, we would like to better investigate the effectiveness of our approach by applying it to further case studies (including industrial applications).

## 5 Related Work

In literature, the operational profile has been recognized as a very relevant factor in many domains, such as software reliability [27] and testing [30]. In the context of performance analysis of software systems, there are many techniques developed to act at: (i) design-time, i.e., providing model-based predictions [6,12,32]; (ii) run-time, i.e., actual measurements derived from system monitoring [10,13,35]. The refactoring, instead, is a more recent research direction, and many issues arise when modifying different system abstractions [3,26,5]. This paper contributes in demonstrating that both performance analysis and refactoring are affected by operational profiles, and in the following we review the related work aimed at pursuing this research direction.

In [22], a method for uncertainty analysis of the operational profile is presented, and the perturbation theory is used to evaluate how the execution rates of software components are affected by changes in the operational profile. Our approach also considers execution rates, but it is intended to support designers in the task of identifying performance-critical scenarios (i.e., when antipatterns occur and their evolution when refactoring actions are applied). In [34], performance antipatterns are used to isolate the problems' root causes, and facilitating their solutions; the TPC-W benchmark showed a relevant increase in the maximum throughput, thus to assess the usefulness of performance antipatterns. However, the choice of representative usage profiles is recognized by the authors as a limitation of the approach, since no directives are given for this scope. Our approach, instead, is intentionally focused on exploiting the performance antipatterns while considering the operational profile space as a first-class citizen of the conducted analysis.

The static technique proposed in [25] detects and fixes performance bugs (i.e., break out of the loop when a given condition becomes true). It is applied to real-world Java and C/C++ applications, and it resulted very promising since a large number of new performance bugs are discovered. Like [34], this approach neglects the operational profile that instead may trigger the presence of further performance problems. As opposite, our goal is to shed the light on the importance of the operational profile space, and our experimentation demonstrates that performance problems and solutions indeed vary across such a space.

In [21], performance anomalies in testing data are detected through a new metric, namely the transaction profile (TP), that is inferred from the testing data along with the queueing network model of the testing system. The key intuition is that TP is independent from the workload, it is sensitive to variations caused by software updates only. Our approach also investigates what are the refactorings that are more responsible of performance issues, along with the characteristics of the operational profile. In fact, refactorings produce regions of the operational profile space that are differently affected, and these differences can be used by the designers in the task of understanding the suitability of a specific design. The work more related to our approach is [28] where sequences of code refactorings (for Java-like programs) are driven by the avoidance of antipatterns (i.e., the BLOB only) and aimed at improving the system security. These refactorings consider the attack surface (i.e., how users/attackers access to software functionalities) as an additional optimization objective. Our approach shares the intuition that antipattern-based refactorings are beneficial for software quality (i.e., performance in our case) and that the operational profile needs to be part of the evaluation, but unlike [28] we target software design abstractions, and we provide a global view of the antipatterns encountered by software systems across their entire operational profile space. A systematic literature review on software architecture optimization methods is provided in [1], but users' operational profiles are neglected. This further motivates our work as promoter of a research line that should foster more attention on the role of users and their effects on the available software resources.

Summarizing, to the best of our knowledge, there is no approach that focuses on how the operational profile affects the performance analysis and refactoring of software systems, and the idea of adopting performance antipatterns for this scope seems to be promising according to our experimentation.

## 6 Conclusion

We presented a novel approach that considers the operational profile space of a system under development as a first class citizen in performance-driven analysis and refactoring of software systems. Performance antipatterns profiles have been used to support designers in the nontrivial task of identifying problematic (from a performance perspective) areas of the operational profile space, and refactoring actions are applied to improve the system performance in such areas. Experimental results confirm the usefulness of the approach, and show how it can be used to evaluate the suitability of a specific design in different regions of the operational profile space.

In addition to the areas of future work mentioned in Section 4.4, we plan to extend our approach with the ability to handle reliability and costs constraints, and thus to support trade-off analysis among multiple quality attributes. Finally, the applicability of the approach could be extended by a portfolio of generic refactoring actions (which need to be feasible with our modelling and analysis techniques), and methods that automate the selection of suitable actions from this portfolio.

## References

[1] Aldeida Aleti, Barbora Buhnova, Lars Grunske, Anne Koziolek, and Indika Meedeniya. Software architecture optimization methods: A systematic literature review. *IEEE Transactions on Software Engineering*, 39(5):658–683, 2012

[2] Aldeida Aleti, Catia Trubiani, André van Hoorn, and Pooyan Jamshidi. An efficient method for uncertainty propagation in robust software performance estimation. *Journal of Systems and Software*, 138:222–235, 2018.

[3] Vahid Alizadeh and Marouane Kessentini. Reducing interactive refactoring effort via clustering-based multi-objective search. In *ASE’18*, pages 464–474, 2018.

[4] Davide Arcelli, Vittorio Cortellessa, and Catia Trubiani. Antipattern-based model refactoring for software performance improvement. In *QoSA’12*, pages 33–42, 2012.

[5] Gabriele Bavota, Andrea De Lucia, Massimiliano Di Penta, Rocco Oliveto, and Fabio Palomba. An experimental investigation on the innate relationship between quality and refactoring. *Journal of Systems and Software*, 107:1–14, 2015.

[6] Simona Bernardi, José Merseguer, and Dorina C. Petriu. Dependability modeling and analysis of software systems specified with UML. *ACM Comput. Surv.,* 45(1):2:1–2:48, 2012.

[7] Gunter Bolch, Stefan Greiner, Hermann De Meer, and Kishor S Trivedi. *Queueing networks and Markov chains: modeling and performance evaluation with computer science applications*. John Wiley & Sons, 2006.

[8] William H Brown, Raphael C Malveau, Hays W McCormick, and Thomas J Mowbray. *AntiPatterns: refactoring software, architectures, and projects in crisis*. John Wiley & Sons, 1998.

[9] Axel Busch, Dominik Fuchss, and Anne Koziolek. Peropteryx: Automated improvement of software architectures. In *ICSA-C’19*, pages 162–165, 2019.

[10] Radu Calinescu, Carlo Ghezzi, Marta Z. Kwiatkowska, and Raffaela Mirandola. Self-adaptive software needs quantitative verification at runtime. *Commun. ACM*, 55(9):69–77, 2012.

[11] Radu Calinescu, Milan Ceska Jr., Simos Gerasimou, Marta Kwiatkowska, and Nicola Paoletti. Efficient synthesis of robust models for stochastic systems. *Journal of Systems and Software*, 143:140–158, 2018.

[12] Radu Calinescu and Shinji Kikuchi. Formal methods @ runtime. In *Monterey Workshop*, pages 122–135. Springer, 2010.

[13] Radu Calinescu and Marta Kwiatkowska. CADS*: Computer-aided development of self-* systems. In *FASE’09*, pages 421–424. Springer, 2009.

[14] Radu Calinescu, Danny Weyns, Simos Gerasimou, Muhammad Usman Iftikhar, Ibrahim Habli, and Tim Kelly. Engineering trustworthy self-adaptive software with dynamic assurance cases. *IEEE Transactions on Software Engineering*, 44(11), 1039–1069, 2018.

[15] Xi Chen, Zibin Zheng, Qi Yu, and Michael R. Lyu. Web service recommendation via exploiting location and qos information. *IEEE Trans. Parallel Distrib. Syst.*, 25(7):1913–1924, 2014.

[16] Vittorio Cortellessa, Antinisca Di Marco, and Paola Inverardi. *Model-Based Software Performance Analysis*. Springer, 2011.

[17] Vittorio Cortellessa, Antinisca Di Marco, and Catia Trubiani. An approach for modeling and detecting software performance antipatterns based on first-order logics. *Software and Systems Modeling*, 13(1):391–432, 2014.

[18] Christian Dehnert, Sebastian Junges, Joost-Pieter Katoen, and Matthias Volk. A storm is coming: A modern probabilistic model checker. In *Computer Aided Verification*, pages 592–600. Springer International Publishing, 2017.

[19] Michalis Famelis and Marsha Chechik. Managing design-time uncertainty. *Software and Systems Modeling*, 18(2):1249–1284, 2019.

[20] Simos Gerasimou, Radu Calinescu, and Giordano Tamburrelli. Synthesis of probabilistic models for quality-of-service software engineering. *Autom. Softw. Eng.*, 25(4):785–831, 2018.

[21] Shadi Ghaith, Miao Wang, Philip Perry, Zhen Ming Jiang, Patrick O’Sullivan, and John Murphy. Anomaly detection in performance regression testing by transaction profile estimation. *Softw. Test., Verif. Reliab.,* 26(1):4–39, 2016.

[22] Sunil Kamavaram and Katerina Goseva-Popstojanova. Sensitivity of software usage to changes in the operational profile. In *Annual Workshop of NASA Goddard Software Engineering*, pages 157–164, 2003.

[23] Arijit Khan, Xifeng Yan, Shu Tao, and Nikos Anerousis. Workload characterization and prediction in the cloud: A multiple time series approach. In *NOMS’12*, pages 1287–1294, 2012.

[24] M. Kwiatkowska, G. Norman, and D. Parker. PRISM 4.0: Verification of probabilistic real-time systems. In *CAV’11*, volume 6806 of *LNCS*, pages 585–591, 2011.

[25] Adrian Nistor, Po-Chun Chang, Cosmin Radoi, and Shan Lu. Caramel: Detecting and fixing performance problems that have non-intrusive fixes. In *ICSE’15*, pages 902–912, 2015.

[26] Ali Ouni, Marouane Kessentini, Mel Ó Cinnéide, Houari A. Sahraoui, Kalyanmoy Deb, and Katsuro Inoue. MORE: A multi-objective refactoring recommendation approach to introducing design patterns and fixing code smells. *Journal of Software: Evolution and Process*, 29(5), 2017.

[27] Süleyman Özekici and Refik Soyer. Reliability of software with an operational profile. *European Journal of Operational Research*, 149(2):459–474, 2003.

[28] Sebastian Ruland, Géza Kulcsár, Erhan Leblebici, Sven Peldszus, and Malte Lochau. Controlling the attack surface of object-oriented refactorings. In *FASE’18*, pages 38–55, 2018.

[29] Martina De Sanctis, Catia Trubiani, Vittorio Cortellessa, Antinisca Di Marco, and Mirko Flamminj. A model-driven approach to catch performance antipatterns in ADL specifications. *Information & Software Technology*, 83:35–54, 2017.

[30] Carol Smidts, Chetan Mutha, Manuel Rodríguez, and Matthew J Gerber. Software testing with an operational profile: Op definition. *ACM Computing Surveys (CSUR)*, 46(3):39, 2014.

[31] Connie U. Smith and Lloyd G. Williams. Software performance antipatterns for identifying and correcting performance problems. In *CMG’12*, 2012.

[32] Mirco Tribastone, Stephen Gilmore, and Jane Hillston. Scalable differential analysis of process algebra models. *IEEE Trans. Software Eng.*, 38(1):205–219, 2012.

[33] Kishor S. Trivedi and Andrea Bobbio. *Reliability and Availability Engineering - Modeling, Analysis,and Applications*. Cambridge University Press, 2017.

[34] Alexander Wert, Jens Happe, and Lucia Happe. Supporting swift reaction: automatically uncovering performance problems by systematic experiments. In *ICSE’13*, pages 552–561, 2013.

[35] Xiao Yu, Shi Han, Dongmei Zhang, and Tao Xie. Comprehending performance from real-world execution traces: Adevice-driver case. In *ACM SIGPLAN Notices*, volume 49, pages 193–206, 2014.

